# Appropriate Blood Pressure in Cerebral Aneurysm Clipping for Prevention of Delayed Ischemic Neurologic Deficits

**DOI:** 10.1155/2020/6539456

**Published:** 2020-04-01

**Authors:** Cattleya Thongrong, Pornthep Kasemsiri, Pichayen Duangthongphon, Amnat Kitkhuandee

**Affiliations:** ^1^Department of Anesthesiology, Faculty of Medicine, Khon Kaen University, Khon Kaen, Thailand; ^2^Khon Kaen Head and Neck Oncology Research, Faculty of Medicine, Khon Kaen University, Khon Kaen, Thailand; ^3^Skull Base Surgery Unit, Department of Otorhinolaryngology, Faculty of Medicine, Khon Kaen University, Khon Kaen, Thailand; ^4^Neurosurgery Unit, Department of Surgery, Srinagarind Hospital, Faculty of Medicine, Khon Kaen University, Khon Kaen, Thailand; ^5^The Center of Excellence of Neurovascular Intervention and Surgery, Faculty of Medicine, Khon Kaen University, Khon Kaen, Thailand

## Abstract

**Background:**

Delayed ischemic neurologic deficit (DNID) is a problem after cerebral aneurysm clipping. Intraoperative hypotension seems to be indicated as a risk factor, but it remains a controversial issue with varying low-blood pressure levels accepted.

**Methods:**

A retrospective, hospital-based, case-control study was performed with patients who received general anesthesia for cerebral aneurysm clipping. 42 medical record charts were randomly selected and matched 1 : 2 (1 case with DNID : 2 controls without DNID) based on the type of general anesthetic techniques and severity of subarachnoid hemorrhage. The optimal cutoff points of hemodynamic response were calculated by the area under the curve.

**Results:**

Data suggested that the optimal cutoff points for lowest blood pressure for prevention of DNID should be systolic blood pressure (SBP) of 95 mmHg (sensitivity of 78.6%; specificity of 53.6%), diastolic blood pressure (DBP) of 50 mmHg (sensitivity of 71.4%; specificity of 67.9%), and mean arterial pressure (MAP) of 61.7 mmHg (sensitivity of 85.7%; specificity of 35.7%). Furthermore, the optimal cutoff point mean difference baseline blood pressure was recommended as Δ SBP of 36 mmHg (sensitivity of 85.7%; specificity of 60.7%), Δ DBP of 27 mmHg (sensitivity of 92.9%; specificity of 71.4%), and Δ MAP of 32 mmHg (sensitivity of 92.9%; specificity of 85.7%). No significant difference between DNID and non-DNID groups was found for end-tidal carbon dioxide (ETCO_2_) and has poor diagnostic value for predicting DNID.

**Conclusion:**

To prevent DNID, we recommend that optimal blood pressure should not be lower than 95 for SBP, 50 for DBP, and 61.7 mmHg for MAP. Additionally, we suggest that Δ SBP, Δ DBP, and Δ MAP should be less than 36, 27, and 32 mmHg, respectively.

## 1. Introduction

Cerebral vasospasm (CVS) is a common problem following subarachnoid hemorrhage. Nearly sixty percent of aneurysmal subarachnoid hemorrhage patients will develop CVS [1]. Furthermore, 20–40% of CVS may progress to delayed ischemic neurologic deficits (DNID) [2–4]. Although the mechanism of developing DNID is not well understood, the endothelial dysfunction, loss of autoregulation, and microvascular thrombosis are considered as the major factors [5]. Regarding the loss of cerebral autoregulation, the cerebral blood flow may depend on blood pressure; thus, the lower blood pressure is the contributing factor that leads to depletion of cerebral blood supply and developed DNID. Maintaining adequate blood pressure is very important for avoiding sequalae DNID. However, recommendations for the target point of appropriate blood pressure during surgery vary [2]. However, in contrast to that mentioned above, a recent retrospective observational study found that hypotension and hypertension during aneurysm occlusion were not associated with a poor neurological outcome in aneurysmal subarachnoid hemorrhage patients [6]; therefore, this issue remains controversial. In addition, hypocapnia is another factor that may induce cerebral vasoconstriction and causing brain ischemia [7]. Several studies show that hypocapnia is associated with a poor neurological outcome in traumatic brain injury [8, 9], but the evidence is lacking for aneurysmal subarachnoid hemorrhage patients.

This study was, therefore, conducted to investigate the association between intraoperative hemodynamic parameters and DNID in subarachnoid hemorrhage patients who underwent cerebral aneurysm clipping. The optimal target range of hemodynamic parameters was analyzed to prevent DNID. These results may guide the physician to control and adjust parameters towards achieving excellent neurological outcomes.

## 2. Materials and Methods

This study used a hospital-based retrospective case-control design. Participants were patients selected from those who had received general anesthesia for cerebral aneurysm clipping at Srinagarind Hospital, Khon Kaen University, Thailand, between January 2013 and November 2018. A sample size of 42 subjects was chosen using simple random sampling with 1  : 2 matching (1 case with DNID : 2 controls without DNID) based on the type of general anesthetic techniques used (sevoflurane inhalation base, intravenous propofol base, or mixed technique) and severity of subarachnoid hemorrhage (SAH) following the World Federation of Neurological Societies (WFNS) and Fisher grading scales (FS). This sample size was deemed appropriate considering 95% confidence intervals, 2% error, and meaningful of the area under the curve of 0.76, calculated from the pilot study. Thus, medical record charts were required for 14 patients with DNID and 28 patients without DNID. Complete medical record charts of patients who underwent cerebral aneurysm clipping were enrolled; however, inadequate record charts that did not clearly indicate the severity of SAH and had insufficient hemodynamic response recording were excluded. Regarding blood pressure measurement, the oscillometric device was used for monitoring and it recorded every five minutes; however, capnogram in the anesthesia machine was used for end-tidal carbon dioxide (ETCO_2_) measurement and recorded every fifteen minutes. The lowest point of blood pressure and ETCO_2_ was calculated from the average of three measurements including the lowest value and before and after the lowest value. The study was reviewed and approved by the Khon Kaen University Ethics Committee for Human Research (HE621246).

Fisher's Exact Probability test was used to compare baseline characteristics between patients with and without DNID, including differences in gender, underlying disease, the severity of SAH, and general anesthetic techniques. The Mann–Whitney *U* test was used for analyzing other parameters including estimated blood loss, fluid, medical resuscitation, and hemodynamic response. Cutoff points of the hemodynamic response to prevent DNID were calculated by receiver operating characteristic (ROC) curves using STATA (v 14.0: Stata Corp. 2015, Texas, USA).

## 3. Results

Patient demographics are presented in Table 1. Patients in both groups appear similar except for body weight and estimated intraoperative blood loss that was significantly higher in the DNID group (*p* < 0.05). Before the procedure, the DNID group had significantly higher mean blood pressure (*p* < 0.05), whereas the mean ETCO_2_ was lower in patients without DNID. During the procedure, the mean low-blood pressure points and ETCO_2_ were higher in the DNID group (*p* > 0.05). The mean difference between the lowest blood pressure point during operation and baseline blood pressure point and ETCO_2_ showed a significantly wider range of blood pressure in the DNID group (*p* < 0.05), whereas a narrower range of ETCO_2_ was observed in the DNID group (*p* > 0.05) (Table 2).

**Figure 1:**
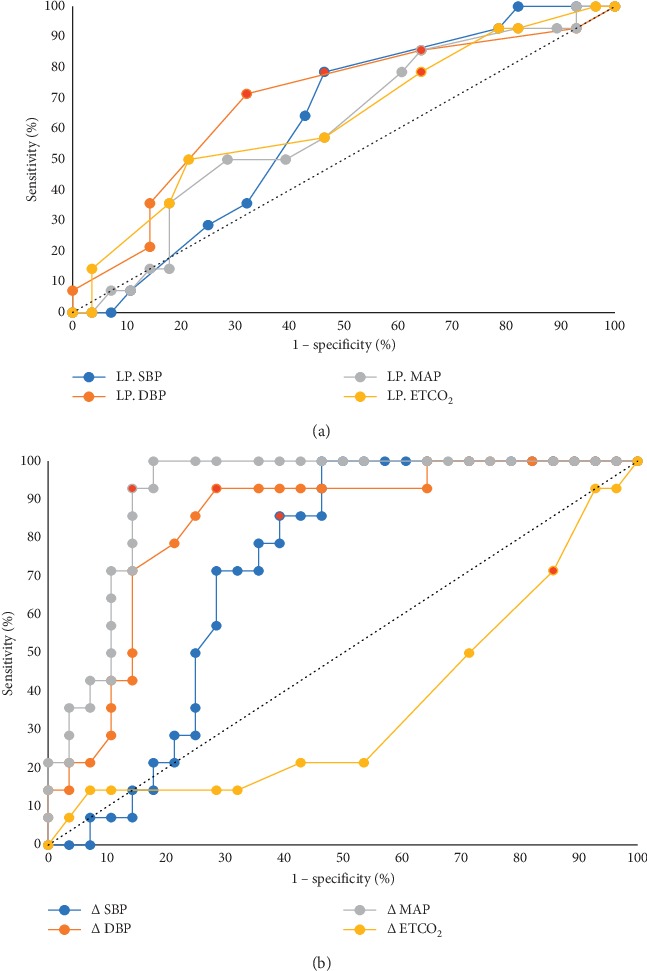
The ROC curve (a) presents the optimized cutoff point of mean of the lowest point of blood pressure and end-tidal carbon dioxide, whereas the optimized cutoff point of the mean difference between the lowest point and the baselined point at initial procedure is shown as the ROC curve. (b) The recommended optimal cutoff point of each line is presented with a red dot (LP, the lowest point; Δ, mean difference; SBP, systolic blood pressure; DBP, diastolic blood pressure; MAP, mean arterial blood pressure; ETCO_2_, end-tidal carbon dioxide).

Recommendations for the optimized cutoff point for prevention of DNID are systolic blood pressure (SBP) of 95 mmHg (sensitivity of 78.6%; specificity of 53.6%), diastolic blood pressure (DBP) of 50 mmHg (sensitivity of 71.4%; specificity of 67.9%), and mean arterial pressure (MAP) of 61.7 mmHg (sensitivity of 85.7%; specificity of 35.7%). These ROC curves of SBP, DBP, and MAP allowed the area under the curve (AUC) of 0.6, 0.7, and 0.6, respectively. In addition, the lowest point of ETCO_2_ is recommended as 27 mmHg (sensitivity of 78.6%; specificity of 35.7%) with AUC of 0.6 (Figure 1(a)).

The mean differences in blood pressure and ETCO_2_ were calculated with the initial operation point and the lowest point during operation. The mean different ROC curves showed that the optimal cutoff points were SBP of 36 mmHg (sensitivity of 85.7%: specificity of 60.7%), DBP of 27 mmHg (sensitivity of 92.9%; specificity of 71.4%), and MAP of 32 mmHg (sensitivity of 92.9%; specificity of 85.7%). The mean different AUC of SBP, DBP, and MAP was 0.7, 0.8, and 0.9, respectively. The optimal cutoff point of the mean difference of ETCO_2_ was 3 mmHg (sensitivity of 71.4%; specificity of 14.3%) with AUC of 0.4 (Figure 1(b)).

## 4. Discussion

The pathogenesis of DNID after SAH is a multifactorial process that leads to neurological deterioration. Therefore, this study was conducted with a matched case-controlled design to reduce confounding factors that may disturb the primary study outcome to determine the optimal range of hemodynamic parameters during cerebral aneurysm clipping. The severity of SAH is concerned that it may be the stimulant factor to develop DNID. The literature showed that 40%–70% of ruptured aneurysms patients with high-grade WFNS developed DNID [10, 11], whereas FS was demonstrated to be associated with DNID of 55% with multivariable analysis and of 62% with univariable analysis [12]. Moreover, our study considered the types of general anesthetic agents as confounding factors; thus, the simple random sampling with 1 : 2 matching based on WFNS, FS, and general anesthetic agents was conducted for patients distribution as 1 case in the DNID group and 2 controls in the non-DNID group that showed no statistically significant difference between both the groups (*p* > 0.99) (Table 1). These results allow balance baseline data between the two groups that facilitate avoiding systematic error and identifying a more accurate optimal target range of hemodynamic parameters and ETCO_2_ for preventing DNID after cerebral aneurysm clipping in SAH patients.

Regarding the hemodynamic response, the relationship between the initial high blood pressure on admission and DNID was unclear. In our study, the initial mean of MAP in the DNID group was higher than that in the non-DNID group (109.5 ± 10.6 vs 89.2 ± 12.2 mmHg; *p* < 0.05), similar to previous studies. For example, Claassen et al. [13] and Frontera et al. [14] reported that elevated initial MAP more than 112 mmHg was a risk factor for the development of DNID. Although the initial SBP and DBP were not reported in the previous studies, we found a significant trend of higher SBP and DBP in the DNID group (152.9 ± 18.3 vs 129.8 ± 25.7; *p* < 0.05 and 87.9 ± 14.0 vs 68.9 ± 10.9; *p* < 0.05, respectively) that may be relative risk factors for DNID.

Higher initial blood pressure in the DNID group seemed related to the mean of the lowest point of blood pressure and the cutoff point of the lowest blood pressure during operation. The mean of the lowest point of blood pressure in the DNID group seemed higher than that in the non-DNID group (*p* > 0.05). For a cutoff point of blood pressure, including SBP, DBP, and MAP, we suggest that blood pressure should be not lower than 95, 50, and 61.7 mmHg, respectively. The lowest point of SBP of 95 mmHg in our study was similar to a previous study that showed SBP lower than 90 mmHg associated with DNID [15]; however, the acceptable lowest point of DBP and MAP were still not agreed in the previous literature.

The mean difference between initial blood pressure and the lowest point of blood pressure during the operation showed better diagnostic value for predicting the effect of initial blood pressure. All of the mean differences in blood pressure were significantly higher statistically in DNID (*p* < 0.05). The ROC curves of mean difference blood pressure provided better AUC (0.7–0.9) than the lowest point of blood pressure (0.6–0.7) and improved the prediction of development of DNID. We suggest the optimal cutoff points of blood pressure below the initial baseline as Δ SBP of 36 mmHg, Δ DBP of 27 mmHg, and Δ MAP of 32 mmHg. In a previous study, Hoff et al. [16] reported that Δ MAP from baseline is associated with poor outcome more than 50% (odds ratio 1.025; 95% CI 1.003–1.047), but this association was declined after adjusting for age and WFNS (odds ratio 1.018; 95% CI 0.996–1.041). Although it did not reach the statistical significance, it seemed clinically meaningful for prediction of development of DNID. The study had a limitation in baseline characteristics of included and not included patients with significantly better clinical condition in the included patients. However, decreasing MAP is still concerning. In our study, we found that decreasing MAP of 32 mmHg or 33.3% from baseline predicted the development of DNID with a sensitivity of 92.9% and specificity of 85.7%, and decreasing SBP from the initial baseline has also been proposed by Chong et al. [17]. They reported Δ SBP of 30 mmHg or 20% from baseline associated with DNID. This finding is similar to our study that showed Δ SBP of 36 mmHg or 26.2% from baseline, allowing sensitivity of 85.7% and specificity of 60.7%. Decreasing DBP from the initial baseline has never been mentioned in previous studies. A cutoff point Δ DBP of 27 mmHg or 35.9% from baseline was observed which provides a sensitivity of 92.9% and specificity of 71.4% in our study. Thus, we suggest that these three blood pressure parameters should be considered together for better prediction of DNID development.

Regarding ETCO_2_, we found no statistically significant difference between the DNID and non-DNID groups (*p* > 0.05). Our ETCO_2_ result was similar to Akkermans et al. [6] that showed no association between ETCO_2_ and the development of DNID with an adjusted risk ratio of 0.95 (95% CI 0.81–1.10; *p* < 0.496) for ETCO_2_ less than 30 mmHg. However, the lowest point of ETCO_2_ in our study is observed at 27 mmHg that yields a sensitivity of 78.6% and specificity of 35.7%. Additionally, the cutoff point of decreasing ETCO_2_ from an initial baseline of 3 mmHg provided a poor diagnostic value with a small AUC of 0.4. Thus, ETCO_2_ may not be a major factor in inducing DNID development.

Regarding the strength of this study, we analyzed the diagnostic value of the optimal blood pressure that allows confident decision making in clinical practice to prevent DNID. Furthermore, the study was conducted with a matched case-controlled design that yielded to reduce confounding factors and balance baseline data. Unfortunately, the limitation of the study was found that some factors including body weight and intraoperative blood loss were significantly higher in the DNID group, though data were insufficient to show that these factors induced DNID. Moreover, the other limitation is its retrospective nature that yields a lack of investigating data including transcranial Doppler, computed tomographic angiogram, and follow-up angiography; therefore, further research is needed to be done in all aspects.

## 5. Conclusion

For preventing the development of DNID, we recommend that the optimal points of blood pressure (including SBP, DBP, and MAP) should not be lower than 95, 50, and 61.7 mmHg, respectively. Additionally, we suggest the mean difference of blood pressure should be less than 36 mmHg for Δ SBP, 27 mmHg for Δ DBP, and 32 mmHg for Δ MAP. However, we found that ETCO_2_ is not a major risk factor associated with DNID.

## Figures and Tables

**Table 1:** Demographic data.

Characteristics	DNID	No DNID	*p* value
Gender, *N* (%)			>0.99
Male	4 (28.6)	9 (32.1)	
Female	10 (71.4)	19 (67.9)	
Age (yrs) mean ± SD	57.4 ± 12.5	56.3 ± 11.4	0.765
Body weight (kg) mean ± SD	64.0 ± 11.0	57.3 ± 9.1	0.042
Height (cm) mean ± SD	160.8 ± 6.2	161.4 ± 5.6	0.751
Hematocrit (%) mean ± SD	38.1 ± 4.6	37.6 ± 6.4	0.825
Underlying disease, *N* (%)			
Diabetes mellitus	1 (3.7)	3 (75.0)	0.250
Hypertension	5 (18.5)	6 (22.2)	0.455
Cardiovascular disease	2 (7.4)	0	0.106
Others	2 (7.4)	8 (29.6)	>0.99
WFNS, *N* (%)			>0.99
1	5 (10.4)	10 (20.8)	
2	3 (6.3)	6 (12.5)	
3	0	0	
4	6 (12.5)	12 (25.0)	
5	0	0	
FS, *N* (%)			>0.99
1	0	0	
2	0	0	
3	6 (12.5)	12 (25.0)	
4	8 (16.7)	16 (33.3)	
General anesthesia technique, *N* (%)			>0.99
Sevoflurane-based	2 (4.2)	4 (8.3)	
Propofol-based	11 (22.9)	22 (45.8)	
Mixed techniques	1 (2.1)	2 (4.2)	
Estimated blood loss (cc)	721.4 ± 352.9	445.4 ± 172.1	0.008
Fluid resuscitation (cc), mean ± SD			
Normal saline	1948.6 ± 739.0	2080.0 ± 723.0	0.406
Colloid	812.5 ± 541.0	464.5 ± 252.2	0.124
Packed red blood cells	2.7 ± 1.1	1.8 ± 0.9	0.045
Fresh frozen plasma	2.2 ± 1.0	1.8 ± 1.3	0.607
Medical resuscitation, mean ± SD			
Ephedrine	18.0 ± 17.0	14.4 ± 8.7	0.925
Levophed	16.0 ± 10.6	26.9 ± 16.3	0.327
Nicardepine	2.5 ± 2.1	0.9 ± 0.2	0.221
Herbesser	5.0 ± 2.7	8.5 ± 5.1	0.332
Esmolol	37.0 ± 19.9	61.7 ± 76.5	0.635
Labetelol	13.3 ± 2.9	45.0 ± 28.3	0.076
Temporary occlusion during surgery, *N* (%)	6 (42.9)	9 (32.1)	0.499
Duration of temporary occlusion (min), mean ± SD	3.8 ± 6.4	1.6 ± 3.4	0.159

**Table 2:** Comparison of intraoperative hemodynamic and end-tidal carbon dioxide monitoring between patients with and without DNID.

Parameters	Overall	Comparison		
		DNID	No DNID	*p* value
At initial operation, mean ± SD				
SBP (mmHg)	137.5 ± 25.7	152.9 ± 18.3	129.8 ± 25.7	<0.05
DBP (mmHg)	75.2 ± 14.9	87.9 ± 14.0	68.9 ± 10.9	<0.05
MAP (mmHg)	96.0 ± 15.1	109.5 ± 10.6	89.2 ± 12.2	<0.05
ETCO_2_ (mmHg)	31.8 ± 4.1	30.2 ± 4.8	32.5 ± 3.6	0.143

During operation				
SBP, mean ± SD				
The lowest point (mmHg)	97.4 ± 12.9	100.4 ± 8.9	95.9 ± 14.5	0.297
Δ (mmHg)	39.0 ± 24.9	49.1 ± 12.8	35.4 ± 26.1	0.020

DBP, mean ± SD				
The lowest point (mmHg)	47.5 ± 7.8	50.7 ± 8.1	45.9 ± 7.2	0.056
Δ (mmHg)	27.7 ± 13.7	37.2 ± 13.4	23.0 ± 11.4	<0.05

MAP, mean ± SD				
The lowest point (mmHg)	65.5 ± 8.1	67.1 ± 7.2	64.7 ± 8.6	0.365
Δ (mmHg)	29.3 ± 13.2	41.2 ± 9.4	23.4 ± 10.7	<0.001

ETCO_2_, mean ± SD				
The lowest point (mmHg)	27.5 ± 2.2	28.2 ± 2.1	27.1 ± 2.2	0.141
Δ (mmHg)	5.1 ± 3.3	4.4 ± 3.7	5.4 ± 3.1	0.139

Δ, mean difference between the lowest point and baselined point at the initial procedure.

## Data Availability

The data used to support the findings of this study are available from the corresponding author upon request.

## Abbreviation

CVS: Cerebral vasospasm
DNID: Delayed ischemic neurologic deficits
SAH: Subarachnoid hemorrhage
WFNS: World Federation of Neurological Societies
FS: Fisher grading scales
ROC: Receiver operating characteristic
LP: Lowest point
Δ: Mean difference
SBP: Systolic blood pressure
DBP: Diastolic blood pressure
MAP: Mean arterial blood pressure
ETCO2: End-tidal carbon dioxide
AUC: Area under the curve.
